# Azvudine versus paxlovid for oral treatment of COVID-19 in Chinese patients

**DOI:** 10.1186/s12879-023-08828-2

**Published:** 2024-01-03

**Authors:** Peng Su, Cong-xian Yang, Xing-guang Wang

**Affiliations:** 1 Department of Hand and Foot Surgery, Shandong Provincial Hospital Affiliated to Shandong First Medical University, Jinan, Shandong China; 2 Department of Respiratory and Critical Care Medicine, Shandong Provincial Hospital Affiliated to Shandong First Medical University, Jinan, Shandong China; 3 Department of Pain Management, Shandong Provincial Hospital Affiliated to Shandong First Medical University, Jinan, Shandong China

**Keywords:** Covid-19, Nirmatrelvir–ritonavir, Paxlovid, Azvudine, Sustained clinical recovery

## Abstract

**Purpose:**

To explore the effect of azvudine as compared to paxlovid for oral treatment of hospitalized patients with SARS-CoV-2 infection.

**Methods:**

We analyzed data from a cohort of patients with SARS-CoV-2 infection in Shandong provincial hospital between February 15 and March 15, 2023. The primary outcome was time to sustained clinical recovery through Day 28 and secondary outcomes included the percentage of participants who died from any cause by Day 28, the average hospitilization time and expenses, the changes in liver and kidney function and adverse events. The Kaplan–Meier method and Cox regression model was used for statistical analysis.

**Results:**

There was no significant difference between azvudine and paxlovid in terms of time to sustained clinical recovery (*p* = 0.429) and death rates (*p* = 0.687). As for hospitalization time and fee, no significant differences were observed between azvudine group and paxlovid group (Hospitalization time: *p* = 0.633; Hospitalization fee: *p* = 0.820). In addition, there were no significant differences in the effects of the two drugs on liver and kidney function (*p* > 0.05).

**Conclusion:**

Among adults who were hospitalised with SARS-CoV-2 infection, azvudine was noninferior to paxlovid in terms of time to sustained clinical recovery, death rates, hospitalization time and cost, with few safety concerns.

**Trial registration:**

ChiCTR2300071309; Registered 11 May 2023.

**Level of evidence:**

Level III; Retrospective cohort study.

## Introduction

The coronavirus disease 2019 (Covid-19) continues to spread rapidly around the world [1, 2], and severe acute respiratory syndrome coronavirus 2 (SARS-CoV-2) has many variants and is becoming more and more infectious [3–5]. Abundant provision of antiviral drugs is crucial for combating the coronavirus [6, 7].

Currently, nirmatrelvir–ritonavir (paxlovid) is recommended by World Health Organization (WHO) guideline for treating mild-to-moderate Covid-19 [8]. Nirmatrelvir is an inhibitor of the main protease Mpro (also known as 3CLpro or nsp5 protease) of SARS-CoV-2, which inhibits its ability to process multi protein precursors, thereby preventing virus replication [9]. Ritonavir is an HIV-1 protease inhibitor and CYP3A inhibitor, which inhibits CYP3A mediated metabolism of nirmatrelvir, thereby increasing the plasma concentration of Nirmatrelvir [10]. The combination of the two drugs can effectively prevent the replication of COVID-19. Remdesivir is also recommended to be administered intravenously for COVID-19, which is inconvenient compared to oral drugs [11]. Therefore, several oral analogues of remdesivir have been developed to address this problem, including GS-621763 [12], ATV006 [13], VV116 [14] and Azvudine [15].

Azvudine is intracellularly converted into a triphosphate form which inhibits the viral RNA-dependent RNA polymerase (RdRp) and has broad-spectrum antiviral activity against hepatitis C virus (HCV) and enterovirus 71 (EV71) as well [16]. A recent study demonstrated that azvudine could cure COVID-19 patients [17]. In China, azvudine was initially developed as a broad-spectrum antiviral agent which obtained emergency use to treat adult COVID-19 patients with moderate symptoms in July 2022 [18]. Paxlovid and azvudine were widely used to respond to the omicron surge in the winter of 2022. However, the efficacy of azvudine for clinical recovery and drug safety remains unknown, particularly as compared to paxlovid. Here, we report the results of a retrospective study of azvudine as compared to paxlovid for oral treatment of hospitalized patients with a confirmed diagnosis of SARS-CoV-2 infection during the omicron outbreak.

## Methods

### Participants

This study was approved by the hospital ethics committee in Shandong Provincial Hospital. The hospital Electronic Medical Record System was searched for records of hospitalized patients diagnosed with Covid-19 treated from February 15, 2023 to March 15, 2023. The inclusion criteria was as below: 1) subjects must be at least 18 years old; 2) subjects have positive SARS-CoV-2 test results; 3) subjects has one or more mild or moderate COVID-19 clinical symptoms, and the symptom score is ≥ 2 points: fever, cough, sore throat, nasal congestion or runny nose, headache, muscle pain, nausea, vomiting, diarrhea, shortness of breath or dyspnea, chills or chills; 4) the first occurrence of COVID-19 symptoms is more than 5 days from the first administration of the trial drug; 5) It is necessary to meet one or more of the following high-risk factors that progress to severe COVID-19 (including death): Age ≥ 60 years old; Coronary heart disease or hypertension; Stroke; Chronic obstructive pulmonary disease; Diabetes; Obesity or overweight BMI > 25 kg/m^2^; Cancer. The exclusion criteria was as below: 1) SpO_2_ ≤ 93% or PaO2/FiO2 ≤ 300, or respiratory rate ≥ 30/min in indoor air at sea level on admission; 2) Mechanical ventilation is required on admission; 3) Subjects have received SARS-CoV-2 monoclonal antibody treatment or preventive or antiviral treatment (including research treatment); 4) Subjects have received COVID-19 plasma treatment in recovery period.

Patients meeting the inclusion criteria were assigned to the test group and the control group. The test group was azvudine. The subjects received azvudine 5 mg qd on day 1 to day 7. The control group was paxlovid. The subjects received nirmatrelvir tablets 300 mg + ritonavir tablets 100 mg, q12h on day 1 to day 5.

### Assessment

When patients were admitted to our hospital for the treatment of Covid-19, a Covid-19-related symptom score scale was given and a dedicated person guided them to fill out the scale. This process continued until day 28 after taking the drug. Covid-19-related symptom score ranged from 0 to 3 for each of 11 symptoms, with higher scores denoting more severe symptoms; When all Covid-19-related target symptoms alleviated to a total symptom score of 0 or 1 for 2 consecutive days, sustained clinical recovery was defined. The first day of the 2-consecutive-day period was considered to be the event date. In addition, patients were routinely checked for alanine transaminase, glutamic acid transaminase and creatinine before and after taking the drug orally to detect the effect of the drug on liver and kidney function.

### End points

The primary efficacy end point was the time to sustained clinical recovery through day 28. The secondary end points included the percentage of the participants who died of any cause by day 28, the average hospitalization time, the average hospitalization expenses, the changes in liver and kidney function during oral drug and adverse events during oral drug.

### Statistical analysis

The Kaplan–Meier method was used to estimate the median time with the 95% confidence interval to sustained clinical recovery and to identify the significant difference of time to sustained clinical recovery. The Cox regression model was used to identify whether oral medication independently affects time to sustained clinical recovery. For the death rate through day 28, chi-square test was used to test the differences between the two drugs. For the average hospitalization time, the average hospitalization expenses and the changes in liver and kidney function during oral drug, independent sample t-test was used to test the differences between the two drugs. The significance level was set at *P*-value < 0.05.

## Results

A total of 171 participants were included in this study. Eighty-eight received azvudine and eighty-three received paxlovid. The characteristics at baseline were balanced between the azvudine group and the paxlovid group. The details were shown in Table 1.

**Table 1:** Demographic and clinical characteristics of the population

Characteristic	Azvudine (*N* = 88)	Paxlovid (*N* = 83)	Total (*N* = 171)
Mean age on admission (range) — yr	70.3 (25–90)	70.1 (36–89)	70.7 (25–90)
Sex—no. (%)			
Male	55 (62.5%)	53 (63.9%)	108 (63.2%)
Female	33 (37.5%)	30 (36.1%)	63 (36.8%)
Vaccination status—no. (%)			
Unvaccinated	42 (47.7%)	27 (32.5%)	69 (40.4%)
Vaccinated	46 (52.3%)	56 (67.5%)	102 (59.6%)
BMI—no. (%)			
BMI ≥ 25	50 (56.8%)	49 (59.0%)	99 (57.9%)
BMI < 25	38 (43.2%)	34 (41.0%)	72 (42.1%)
Risk factors for severe illness from Covid-19 — no. (%)			
Age ≥ 60 yr	76 (86.4%)	68 (81.9%)	144 (84.2%)
Hypertension	40 (45.5%)	31 (37.3%)	71 (41.5%)
Diabetes	26 (29.5%)	9 (10.8%)	35 (20.5%)
Coronary Disease	22 (25.0%)	21 (25.3%)	43 (25.1%)
Stroke	10 (11.4%)	8 (9.6%)	18 (10.5%)
COPD	7 (8.0%)	16 (19.3%)	23 (13.5%)
Malignant Tumor	10 (11.4%)	8 (9.6%)	18 (10.5%)

*Abbreviation*: *yr* year, *BMI* body mass index, *COPD* chronic obstructive pulmonary disease

The mean age of the participants was 71 years old, and 63% were men. Nearly 60% were vaccinated. The most common risk factor for progression to severe Covid-19 at baseline was an age of 60 years or older (84%), followed by a body mass index of 25 or higher (58%), hypertension (42%), cardiovascular disease (26%), diabetes (20%), COPD (13%), stroke (11%) and cancer (11%). All participants received trial regimens beyond 5 days after symptom onset.

### End points

**Fig. 1:**
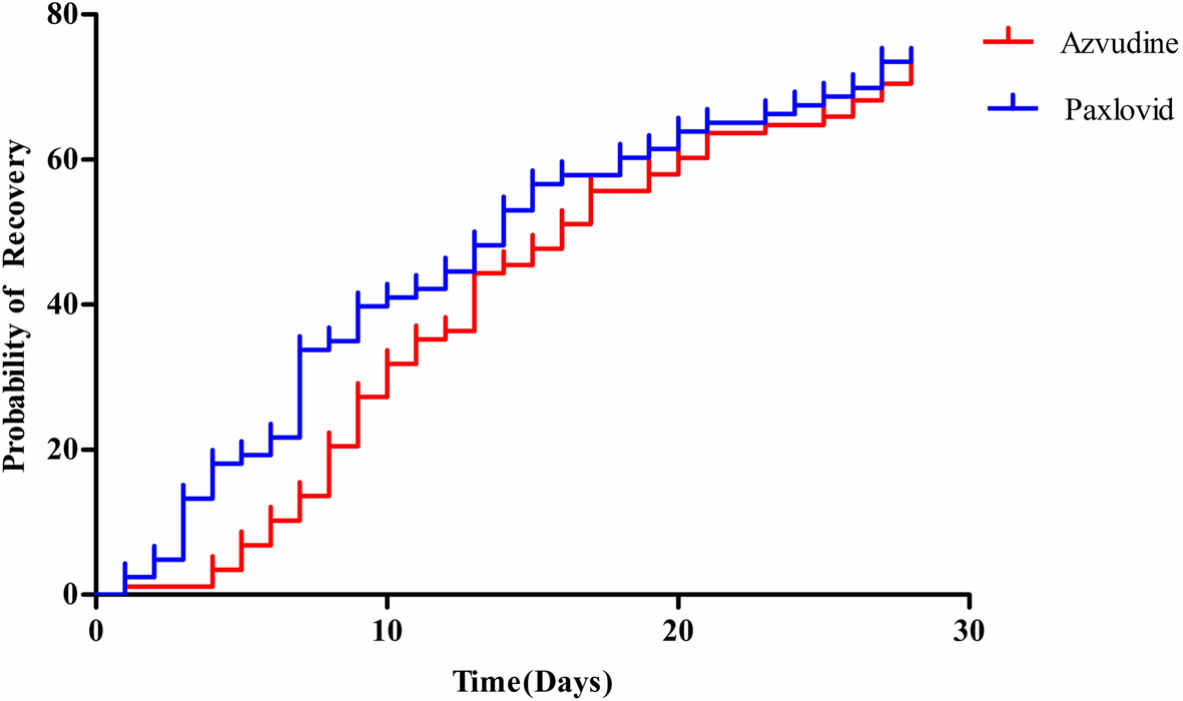
Time to sustained clinical recovery. The results were estimated by means of the Kaplan–Meier method

In the primary analysis, sustained clinical recovery occurred in 64 participants in the azvudine group and 61 participants in the paxlovid group. There was no significant difference between azvudine and paxlovid in terms of time to sustained clinical recovery (Azvudine: median time: 16, 95% confidence interval (CI): 12.3 to 19.7; Paxlovid: median time: 14, 95% confidence interval (CI): 11.2 to 16.7; *p* = 0.429). The details were shown in Fig. 1.

**Fig. 2:**
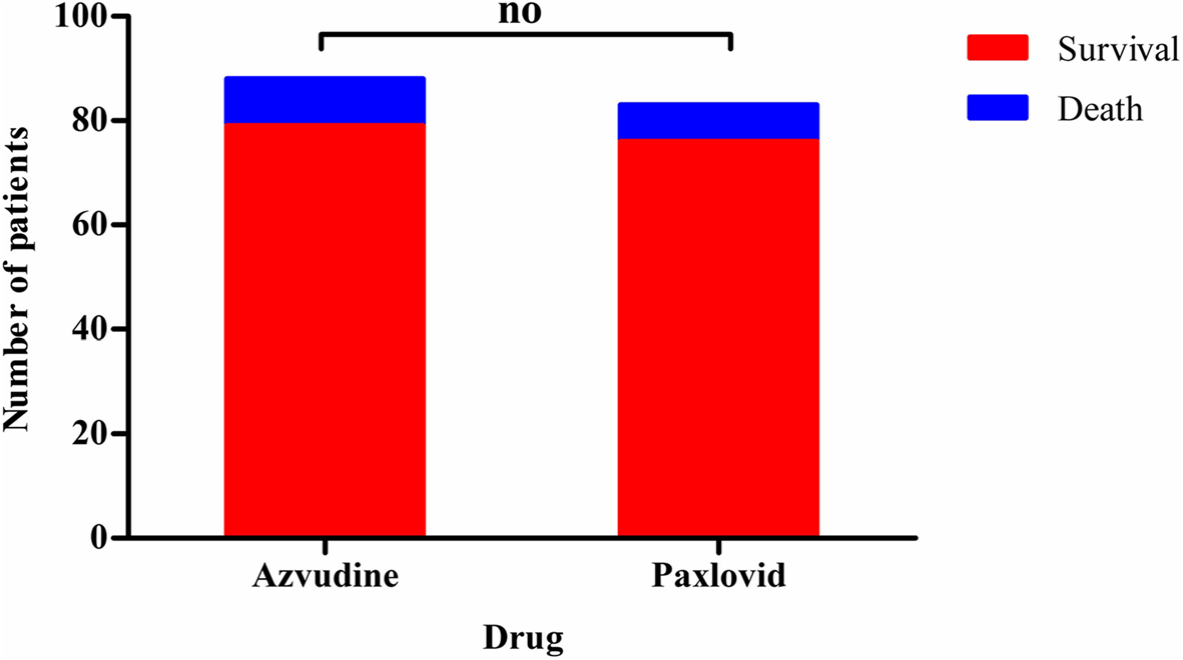
The death rates of both groups by day 28. The results were estimated by ratios of Chi-square test. “no” means “no significant difference” (*p* < 0.05)

As for death rates, 9 participants in azvudine group and 7 participants in paxlovid group had died by day 28. There were no significant difference between the two groups (*p* = 0.687). The details were shown in Fig. 2.

**Fig. 3:**
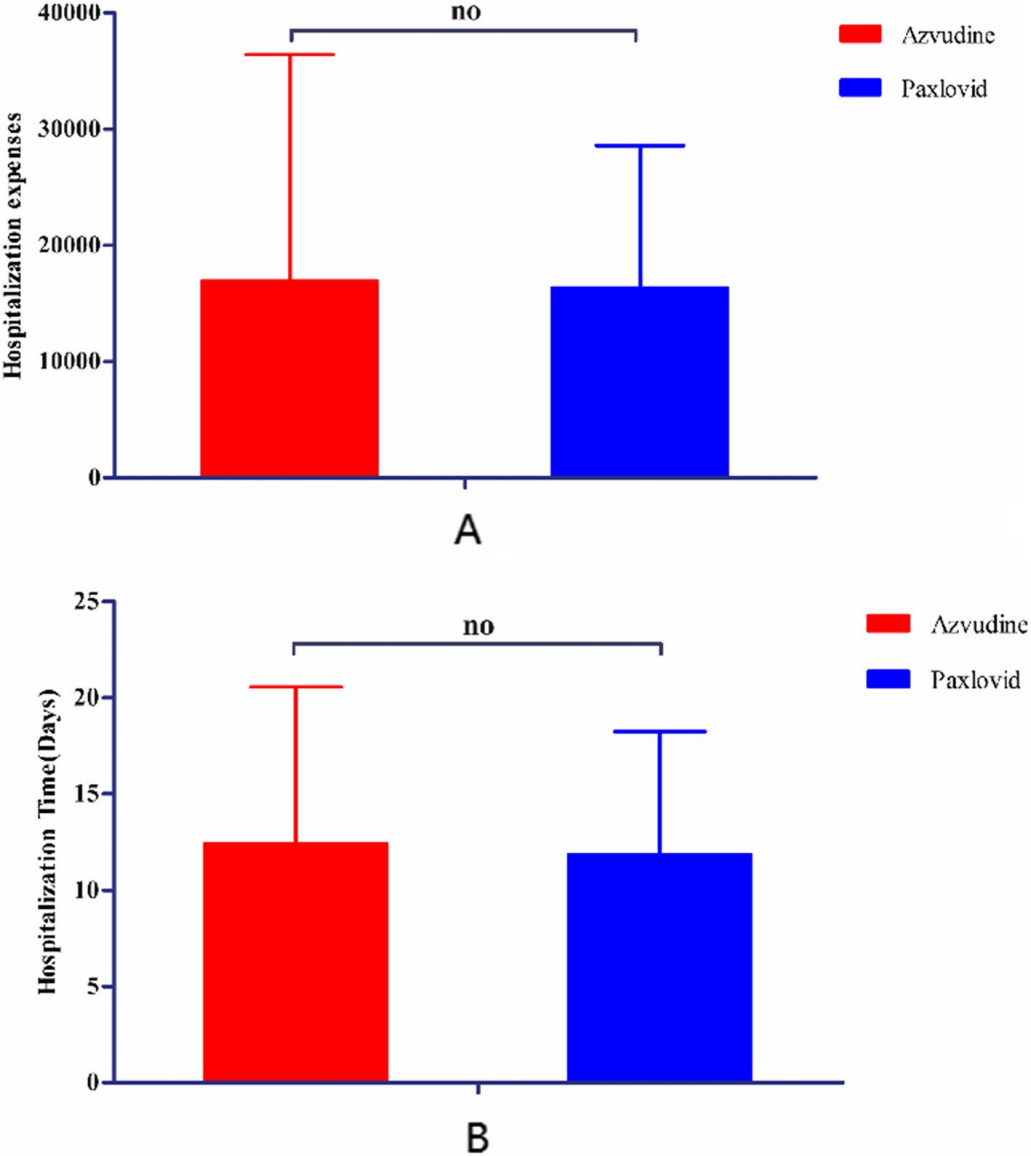
Hospitalization time and expenses of both groups. The unit of the expenses was yuan. The results were estimated by independent-sample t test. “no” means “no significant difference” (*p* < 0.05)

As for hospitalization time and cost, there were no significant differences between the azvudine group and the paxlovid group (Hospitalization time—azvudine: 12.4 ± 8.1, paxlovid: 11.9 ± 6.4; *p* = 0.633; Hospitalization cost—azvudine: 16941.1 ± 19478.0, paxlovid: 16343.8 ± 12233.8; *p* = 0.820). The details were shown in Fig. 3.

As for the effects of the two drugs on liver and kidney function, there were no significant differences in changes before and after oral drugs for Alanine transaminase, glutamic acid transaminase and creatinine between the azvudine group and the paxlovid group (Alanine transaminase: *p* = 0.336; Glutamic acid transaminase: *p* = 0.203; creatinine: *p* = 0.833).

As for adverse events of oral drugs, the most frequently reported was dysgeusia, which was reported in 8 participants receiving paxlovid (9.6%) and 7 participants receiving azvudine (8.0%). The events were not serious. No one withdrew due to adverse events.

In the end, univariate analysis was conducted to investigate whether age, gender, BMI, vaccination status and oral drug type was related with the time to sustained clinical recovery. The results showed that only vaccination status and BMI were associated with time to sustained clinical recovery (vaccination status: *p* = 0.007; BMI: *p* = 0.049). Multivariate Cox regression analysis was then used to investigate whether BMI and vaccination status were associated with time to sustained clinical recovery. The results showed that only vaccination status was associated with time to sustained clinical recovery (*p* = 0.009). This indicated that vaccination status was an independent factor affecting clinical recovery. Vaccinated patients had significantly reduced clinical recovery time.

## Discussion

Although previous research has confirmed that oral azvudine cures COVID-19 patients, with 100% viral ribonucleic acid negative conversion which was indicative of viral clearance [17], the current trial compared azvudine with paxlovid to assess clinical end points and adverse events. This trial showed that in symptomatic adults hospitalized with mild to moderate Covid-19 who were at high risk for severe disease, a 7-day course of oral treatment with azvudine was noninferior to paxlovid in shortening the time to sustained clinical recovery, death rates, hospitalization time and fee, with a strong safety profile. Timely administration can help reduce the burden of hospitalization, minimize household expenses and block potential transmission. In addition, the administration of oral antiviral drugs is relatively convenient.

This trial was conducted during an outbreak of Covid-19 (January and February 2023). The main variant involved in our trial was omicron subvariants BF.7 and BA.5.2 [16]. In this population, the median time to sustained clinical recovery in both groups was longer than that reported in the other trial, where the median time to sustained clinical recovery in the paxlovid group was 5.5 days [19]. The rapid replication period of the virus is from day 1 to day 5 of Covid-19, so it needs to be taken within the first five days. The earlier you take the medicine, the better the effect. In this trial, all patients took the drugs more than 5 days after the onset of infection. This may be the reason for the extension of time to sustained clinical recovery.

In this trial, 60% patients had been vaccinated against SARS-CoV-2. Recent studies have shown that treatment with paxlovid in vaccinated patients with Covid-19 is associated with a reduced risk of hospitalization or progression to severe Covid-19 [20]. This is consistent with the conclusion of this study. In this study, multivariate Cox regression analysis demonstrated that vaccine was associated with time to sustained clinical recovery, and vaccines was an independent factor affecting clinical recovery.

Previous studies have shown that paxlovid has minimal impact on liver and kidney function in patients with SARS-CoV-2 [21]. Currently, there is a lack of evidence of the effect of azvudine on liver and kidney function in patients with SARS-CoV-2. In this study, there were no significant differences in changes before and after oral medication for liver and kidney function, demonstrating that azvudine is also relatively safe for patients with SARS-CoV-2.

As for adverse events of oral drugs, one previous study reported transient dysgeusia in 25% of the participants receiving paxlovid [14]. The other study reported transient dysgeusia in 5.6% of the participants receiving paxlovid [8]. In this study, transient dysgeusia was reported in 9.6% of the participants receiving paxlovid, which proved that transient dysgeusia as as an adverse event was rare.

The incidence rates of transient dysgeusia of azvudine have not been reported so far. This study indicated that transient dysgeusia as an adverse event was less common for azvudine. In this study, no patients discontinued oral drugs due to adverse events, which demonstrated that both drugs were relatively safe for patients with SARS-CoV-2.

The trial has several limitations. First, this study was a retrospective study with a high risk of bias. The difference in the characteristics at baseline between the two groups may also lead to unreliable results. Second, the trial involved Chinese adults infected with omicron subvariants in a single center, so the results need to be validated in a more heterogeneous population with other virus variants. Third, time to sustained clinical recovery was recorded and collected by patients themselves. The data might be very inconsistent.

## Conclusion

Among adults who were hospitalized with a confirmed diagnosis of SARS-CoV-2 infection, azvudine was noninferior to paxlovid in terms of time to sustained clinical recovery, death rates, hospitalization time and cost, with few safety concerns.

## Data Availability

The datasets used and/or analyzed during the current study are available from the corresponding author on reasonable request.
